# Loss of Drosha underlies dopaminergic neuron toxicity in models of Parkinson’s disease

**DOI:** 10.1038/s41419-018-0716-5

**Published:** 2018-06-07

**Authors:** Ronglin Wang, Fangfang Lu, Gang Zhu, Dayun Feng, Tiejian Nie, Kai Tao, Shaosong Yang, Jie Lei, Lu Huang, Zixu Mao, Qian Yang

**Affiliations:** 1 Department of Neurosurgery, Tangdu Hospital, The Fourth Military Medical University, Xi’an, 710038 China; 2 Departments of Pharmacology and Neurology, Emory University School of Medicine, 615 Michael Street, Atlanta, GA 30322 USA; 3 Department of Neurology, GuangZhou Medical University, Guangzhou, Guangdong 510120 China

## Abstract

MiRNAs, a group of powerful modulator of gene expression, participate in multiple cellular processes under physiological and pathological conditions. Emerging evidence shows that Drosha, which controls the initial step in canonical miRNA biogenesis, is involved in modulating cell survival and death in models of several diseases. However, the role of Drosha in Parkinson’s disease (PD) has not been well established. Here, we show that the level of Drosha decreases in 6-OHDA-induced cellular and animal models of PD. 6-OHDA induced a p38 MAPK-dependent phosphorylation of Drosha. This triggered Drosha degradation. Enhancing the level of Drosha protected the dopaminergic (DA) neurons from 6-OHDA-induced toxicity in both in vitro and in vivo models of PD and alleviated the motor deficits of PD mice. These findings reveal that Drosha plays a critical role in the survival of DA neurons and suggest that stress-induced destabilization of Drosha may be part of the pathological process in PD.

## Introduction

Parkinson’s disease (PD) is the most common neurodegenerative disease affecting the motor system. The disease is characterized by the selective loss of dopaminergic (DA) neurons in the substantia nigra pars compacta (SNc)^1^. The precise mechanisms underlying DA neuronal degeneration are complex and remain to be fully illustrated. Many processes including oxidative stress, mitochondrial dysfunction, protein aggregations, and chronic inflammation have all been shown to be involved in its pathogenesis^2,3^. PD pathogenesis is associated with complex changes of signaling events including dysfunction or dysregulation of many kinases^4,5^. Among them, the p38 MAPK is activated by many pathogenic stressors^6,7^.

MiRNAs are a class of small-non-coding RNA. As powerful post-transcriptional gene expression regulators, miRNAs play a critical role in maintaining cellular homeostasis. Recent studies have demonstrated that specific miRNAs contribute to pathogenesis of PD^8–10^. Stresses can alter the biogenesis of miRNAs to affect their function^11^. MiRNA biogenesis involves several tightly coupled sequential steps and is controlled by several protein complexes. Among them, Drosha acts first in the miRNA biogenic cascade to process the conversion of primary (pri)-miRNA to precursor (pre)-miRNA^12–14^. Compared with the understanding about how Drosha processes miRNA, little is known about how Drosha is regulated under physiological and pathological conditions. It is known that post-translational modifications regulate the function and stability of protein factors^15,16^. Our previous study revealed that Drosha is directly phosphorylated by p38 MAPK under stress conditions. Phosphorylation of Drosha by p38 MAPK triggers its degradation, which leads to cell death^17^. However, little is known whether Drosha is targeted by conditions associated with neurodegeneration including PD.

We show in the current study that 6-hydroxydopamine (6-OHDA), a neurotoxin widely used to model PD in vitro and in vivo, causes a p38 MAPK-dependent phosphorylation of Drosha, leading to its dysfunction. Importantly, restoring the level of Drosha protected the SNc DA neurons and alleviated the motor deficits in a mouse model of PD. These findings suggest that loss of Drosha may underlie in part the vulnerability of the SNc DA neurons to pathogenic stress and contribute to their selective loss in PD.

## Results

### 6-OHDA reduced the stability of Drosha in a mouse model of PD

**Fig. 1:**
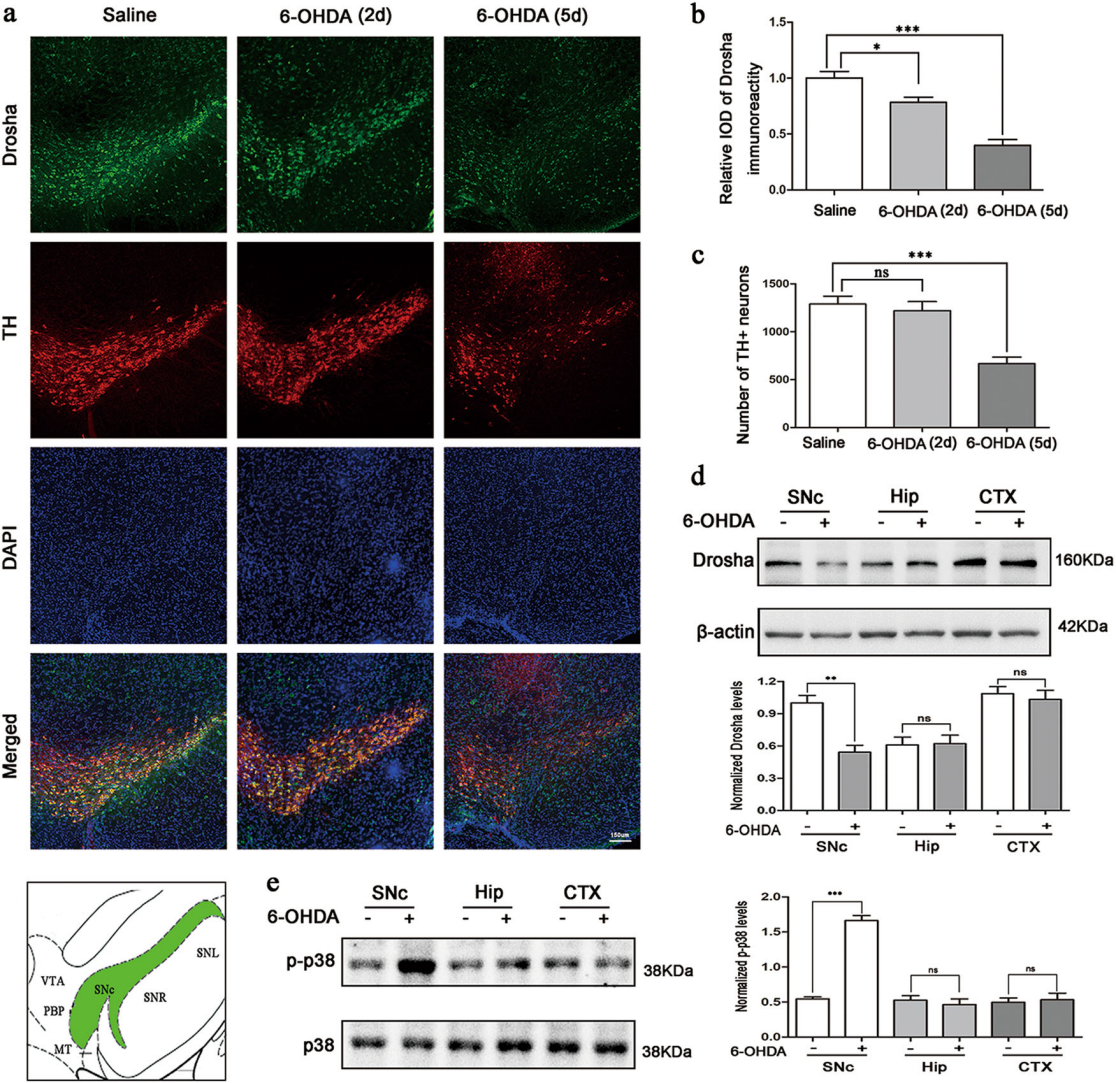
6-OHDA reduced the stability of Drosha in a mouse model of PD. **a** High panels: Drosha levels and TH-positive DA neurons in SNc of saline control mice and 6-OHDA lesioned PD mice. Saline or 0.3 ul 6-OHDA (20 μM) was injected into the SNc of mouse brain. Five days after injection, the brains were perfused with 0.9% NaCl solution and cold 4% paraformaldehyde in phosphate buffer. Then the brains were removed for immunofluorescence. The dilution ratio of Drosha was 1:100 and TH was 1:1000 (*n* = 3). Lower panels: The position of SNc in the midbrain. **b** The quantitative value of Drosha. (ANOVA test followed by Tukey HSD, *P < 0.05, ***P < 0.001, *n* = 3). **c** The number of TH-positive neurons. (ANOVA test followed by Tukey HSD, ***P < 0.001, *n* = 3). **d** Western blot analysis of Drosha level in different brain regions. Five days after injection, the brains were perfused with 0.9% NaCl solution and removed for immunoblot. An anti-Drosha antibody was used to determine the level of Drosha at a dilution ratio of 1:500. An anti-β-actin antibody was used as a loading control. The data are expressed as mean ± S.E.M. (Student’s *t*-test, **P < 0.01, *n* = 3). **e** Western blot analysis of p-p38 level in different brain regions. The brain was removed for immunoblot 2 days after injection. The data are expressed as mean ± S.E.M. (Student’s *t*-test, **P < 0.001, *n* = 3)

Studies have shown that cellular stress regulates the stability of Drosha^17^. To test whether neurotoxins associated with PD can modulate Drosha in PD, we injected 6-OHDA into the SNc to induce stress and the loss of DA neurons, a widely used in vivo model of PD^18^. At 2 and 5 days after injection, we analyzed the midbrain sections by immunofluorescence. The results showed that 6-OHDA reduced Drosha level in TH-positive DA neurons after 2 days while the number of TH positive neurons remained unchanged. At 5 days after injection, the level of Drosha and number of DA neurons all decreased in PD mice midbrain (Fig. 1a–c). Immunoblotting analysis showed that the Drosha level is greatly reduced in the SNc at 5 days after 6-OHDA. In contrast to the SNc region, the level of Drosha in the cortex (CTX) and hippocampus (Hip) regions were not significantly altered (Fig. 1d). Stress kinase p38 has been reported to be activated in the presence of neurotoxin^19^. The Western blot analysis verified a robust increase of p-p38 in the SNc region at 2 days after neurotoxin injection (Fig. 1e). Together, these results indicate that 6-OHDA activates p38 and reduces the stability of Drosha in the mouse SNc region.

### 6-OHDA led to a p38 MAPK dependent phosphorylation of Drosha

**Fig. 2:**
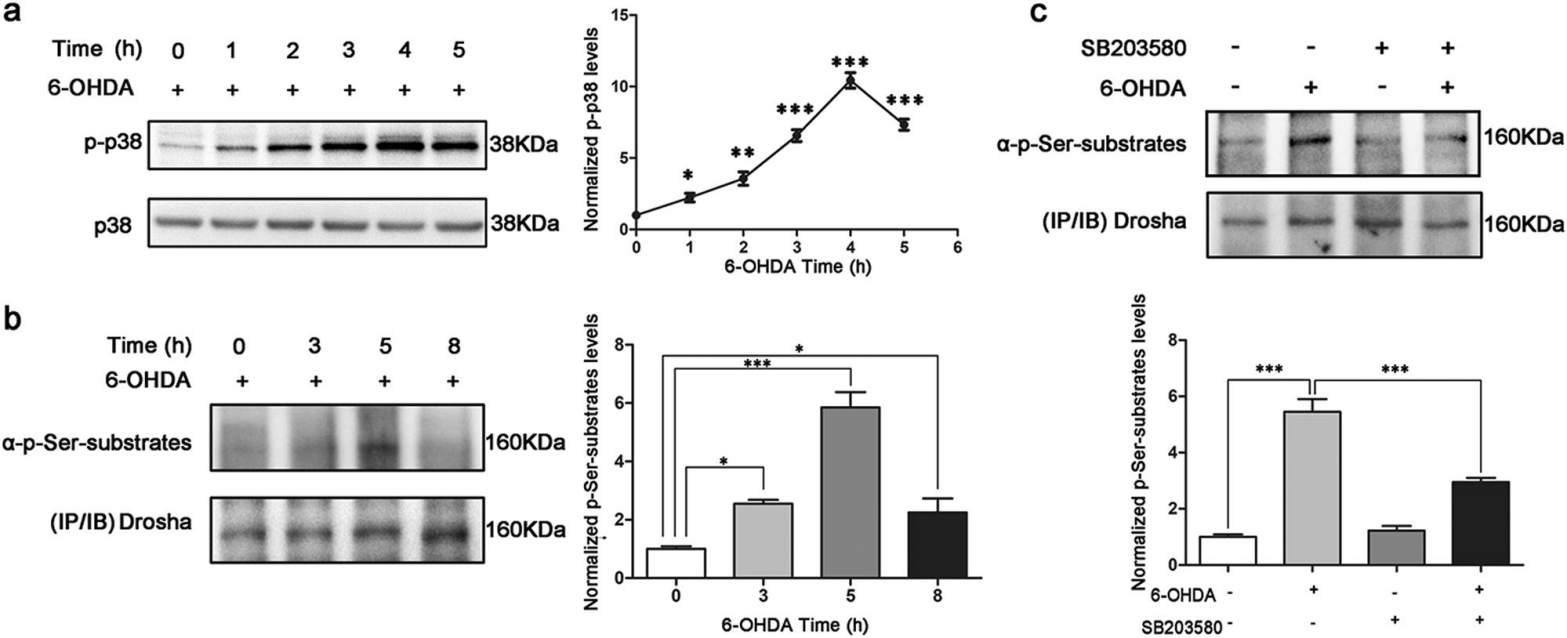
6-OHDA caused a p38 MAPK dependent phosphorylation of Drosha. **a** p38 MAPK was activated with the treatment of 6-OHDA. SN4741 cells were treated with 40 μM 6-OHDA in indicated time. The cell lysate was analyzed by Western blot using an antibody against phospho p38 (9211). An anti-p38 antibody was used as a loading control. Densitometric analyses of the western blots are shown as curves. Data represent mean ± S.E.M. of three independent experiments (ANOVA test followed by Tukey HSD, *P < 0.05, **P < 0.01, ***P < 0.001, *n* = 3). **b** Phosphorylation of Drosha when treated with 6-OHDA for various time course. SN4741 cells were treated with 40 μM 6-OHDA for indicated time. The cell lysate were subjected to immunoprecipitation using an anti-Drosha antibody. The cell lysate should incubate with the antibody for more than 16 h at 4 ℃. An anti-phospho S/P antibody was used to detect the phospho Ser signal at Drosha position. The same membrane was reprobed with anti-Drosha antibody. We try to make the total level of Drosha to be same for it’s more convenient to compare the phospho Ser signal among various groups. (ANOVA test followed by Tukey HSD, *P < 0.05, ***P < 0.001, *n* = 3). **c** The phosphorylation of Drosha is blocked by p38 MAPK inhibitor when exposed to 6-OHDA. 20 μM SB203580 was added to SN4741 cells half an hour before utilization of 40 μM 6-OHDA for 5 h. (ANOVA test followed by Tukey HSD, ***P < 0.001, *n* = 3)

Since our previous studies showed that p38 MAPK directly phosphorylates Drosha under oxidative stress, leading to Drosha degradation, we tested whether p38 MAPK is involved in the modulation of Drosha by 6-OHDA in cellular model^17^. We treated SN4741 cells, a mouse midbrain DA progenitor cell line, with 6-OHDA and analyzed the activity of p38 MAPK. The results showed that 6-OHDA caused a time dependent activation of p38 MAPK in SN4741 cells and p-p38 peaked at about 4 h after treatment, consistent with previous study (Fig. 2a)^20^. Our group have identified the presence of several MAPK phosphorylation sites at the N-terminal of Drosha (Fig. 4a)^17^. To explore whether Drosha was phosphorylated under 6-OHDA stress, we immunoprecipitated endogenous Drosha from SN4741 cells following 6-OHDA treatment and blotted the precipitates with an antibody that specifically recognizes proline-directed phosphorylated serine. Compared with the control group, treatment of 6-OHDA led to a time-dependent increase in phospho Ser signal migrating at Drosha position (Fig. 2b and S1a). To determine the role of p38 MAPK in Drosha phosphorylation, p38 inhibitor SB203580 was added to SN4741 cells half an hour before the 6-OHDA treatment. This analysis showed that SB203580 reduced 6-OHDA-induced phosphorylation of Drosha (Fig. 2c and S1b). Thus, these data indicate that p38 MAPK is directly involved in phosphorylating Drosha in response to 6-OHDA.

### p38 MAPK engaged in the degradation of Drosha induced by 6-OHDA

**Fig. 3:**
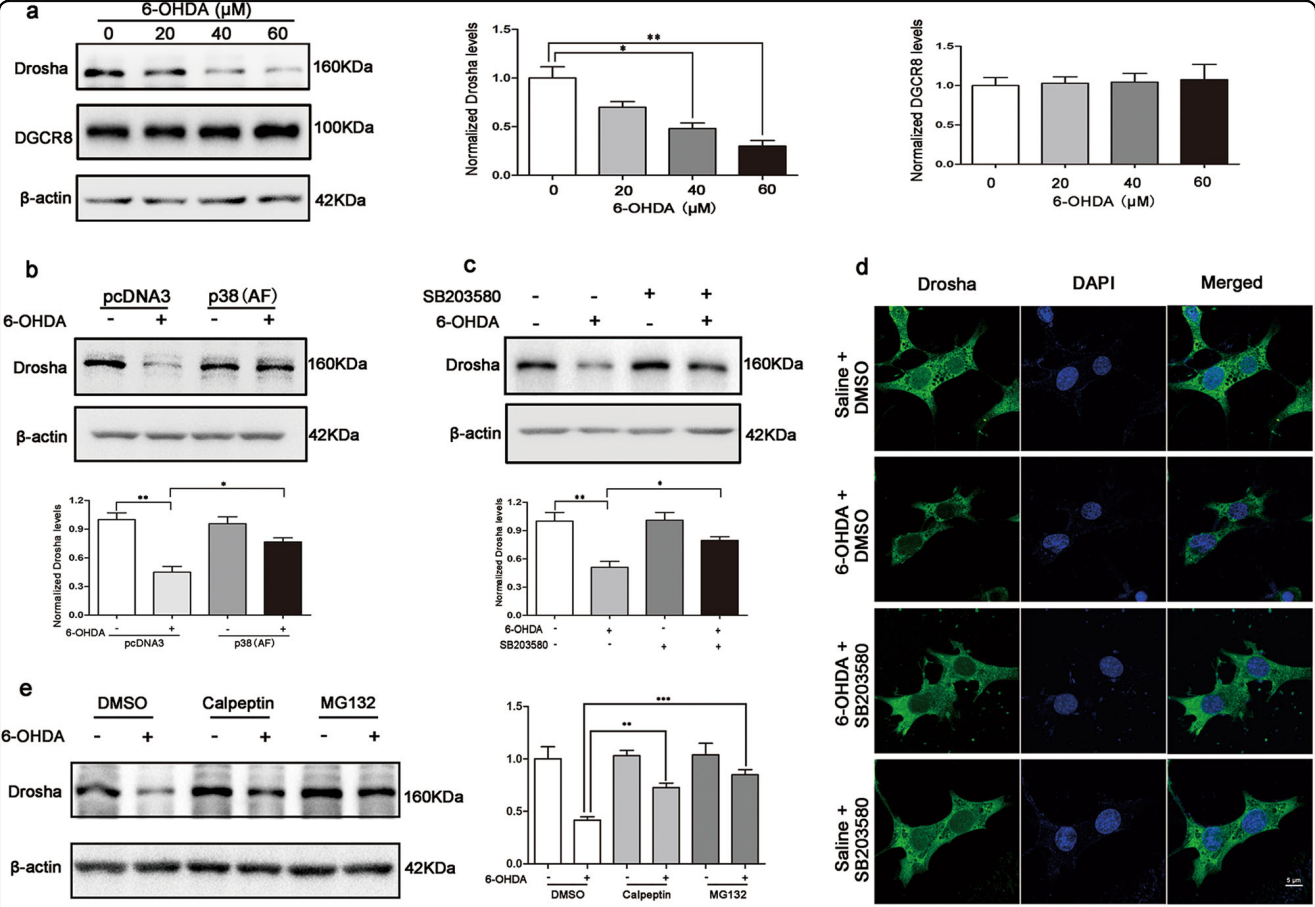
p38 MAPK engaged in the degradation of Drosha induced by 6-OHDA **a** Western blot analysis of Drosha and DGCR8 level in SN4741 cells when applied with 6-OHDA. SN4741 cells was treated with 20, 40, and 60 μM 6-OHDA for 12 h. Anti-Drosha and anti-DGCR8 antibodies were used to detect the protein levels. Densitometric values were normalized using β-actin as an internal control. (ANOVA test followed by Tukey HSD, *P < 0.05, **P < 0.01, *n* = 3). **b** 6-OHDA-induced reduction of Drosha was reversed by transfection of p38 (AF), a kinase dead p38. P38 AF was transfected for 24 h. Then the cells were treated with 40 μM 6-OHDA for 12 h. (ANOVA test followed by Tukey HSD, *P < 0.05, **P < 0.01, *n* = 3). **c** Inhibition of p38 MAPK by SB203580 protected Drosha from 6-OHDA-induced degradation. 20 μM SB203580 was added to SN4741 cells for half an hour before 6-OHDA utilization as described in (**b**). (ANOVA test followed by Tukey HSD, *P < 0.05, **P < 0.01, *n* = 3). **d** The immunofluorescence analysis of SN4741 cells after application of SB203580 and 6-OHDA described in (**c**). **e** Calpeptin and MG132 reverse the decline of Drosha induced by 6-OHDA. 10 uM calpeptin and 5 uM MG132 were applied on SN4741 cells, respectively, for 1 h before 6-OHDA treatment. (ANOVA test followed by Tukey HSD, **P < 0.01, ***P < 0.001, *n* = 3)

To investigate the role of p38 MAPK in 6-OHDA-induced Drosha degradation, we established a prolonged 6-OHDA exposure model. We treated SN4741 cells with 6-OHDA for 12 h at different doses and measured Drosha and its cofactor DGCR8 (DiGeorge syndrome critical region 8) levels by Western blot analysis. The results showed that 6-OHDA treatment caused a dose-dependent gradual decline of Drosha protein level while had little effect on DGCR8 level (Fig. 3a). To examine whether p38 MAPK was engaged in the degradation of Drosha, we transfected SN4741 cells with a kinase dead p38 (AF) construct for 24 h before 6-OHDA treatment. Analysis of Drosha showed that p38 (AF) prevented the reduction of Drosha induced by 6-OHDA (Fig. 3b). To corroborate with this finding, we pretreated SN4741 with SB203580 as indicated. The Western blot analysis and fluorescence analysis showed that inhibition of p38 MAPK protected Drosha from 6-OHDA-induced degradation (Fig. 3c, d). To explore the molecular mechanism responsible for the Drosha degradation, we applied calpain inhibitor calpeptin and ubiquitin proteasome inhibitor MG132 on SN4741 cells before 6-OHDA treatment. The Western blot analysis indicated that both calpeptin and MG132 reversed the decline of Drosha level induced by 6-OHDA (Fig. 3e), indicating that calpain and ubiquitin proteasome are involved in the degradation process of Drosha. The above data shows that p38 MAPK destabilizes Drosha in SN4741 cells upon neurotoxin stress.

### Overexpression of Drosha protected SN4741 cells from 6-OHDA-induced toxicity

**Fig. 4:**
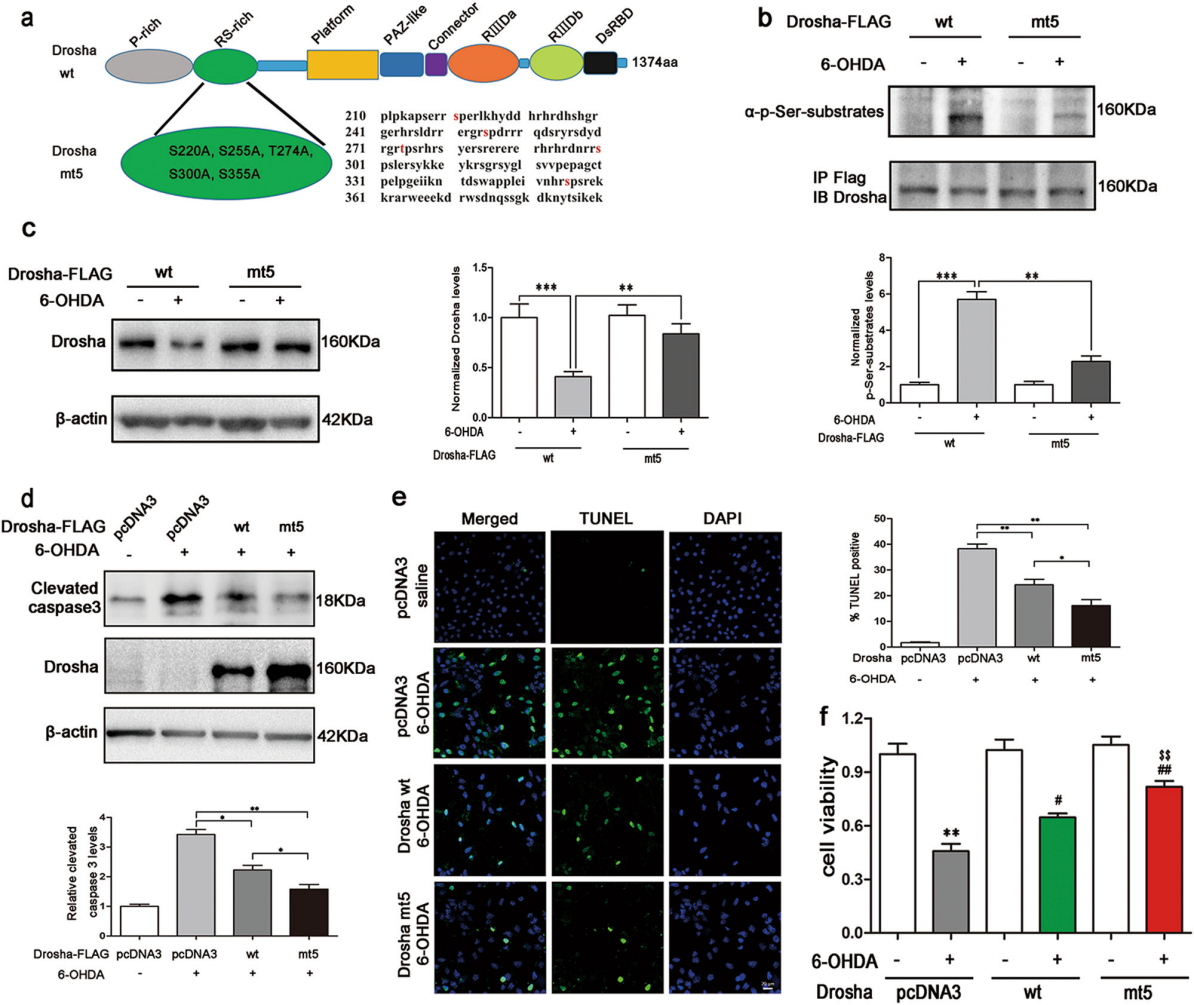
Overexpression of Drosha protected SN4741 cells from 6-OHDA-induced toxicity. **a** The structure of WT and mt5 Drosha. There are five mutations in the RS-rich domain of mt5 Drosha compared to WT Drosha including S220A, S255A, T274A, S300A, and S355A. **b** mt5 Drosha was more resistant to the phosphorylation induced by 6-OHDA. WT and mt5 Drosha were transfected for 24 h in SN4741 cells before application of 40 μM 6-OHDA for 5 h. The cell lysate was managed as described in Fig. 2(a). (ANOVA test followed by Tukey HSD, **P < 0.01 ***P < 0.001, *n* = 3). **c** mt5 Drosha was more resistant to 6-OHDA-induced degradation. After transfection of WT and mt5 Drosha for 24 h, SN4741 cells were treated with 40 μM 6-OHDA for 12 h. (ANOVA test followed by Tukey HSD, **P < 0.01 ***P < 0.001, *n* = 3). **d**–**f** Enhancing Drosha ameliorated the toxicity of 6-OHDA to SN4741 cells and mt5 Drosha offered better protection than WT Drosha. After transfection, SN4741 cells were treated with 40 μM 6-OHDA for 36 h. Clevated caspase3, MTT, and TUNEL assays were used to determine the cellular viability. Densitometric values were normalized using β-actin as an internal control. (ANOVA test followed by Tukey HSD, *P < 0.05, **P < 0.01, ^#^*P* < 0.05, ^##^*P* < 0.01 vs. pcDNA3 treated with 6-OHDA; and ^$$^*P* < 0.01 vs. WT Drosha group treated with 6-OHDA, *n* = 3)

Previously we found that the RS-rich domain of Drosha harbors five p38 MAPK phosphorylation sites (S220, S255, T274, S300, and S355). To verify if p38 MAPK phosphorylated these sites upon 6-OHDA treatment, we tested a Drosha mutant (mt5 Drosha) with all the five sites changed to alanine (Fig. 4a). SN4741 cells were transfected with wide type (wt) or mt5 Drosha 24 h before application with 6-OHDA. After 5 h 6-OHDA treatment, we immunoprecipitated Drosha to examine the phospho Ser signal migrating at Drosha position. The analysis showed that wt Drosha was phosphorylated after 6-OHDA treatment while the mt5 Drosha was more resistant to the phosphorylation (Fig. 4b and S1c). Consistent with this, compared with wt Drosha, mt5 Drosha was much more resistant to 6-OHDA-induced reduction (Fig. 4c).

Prolonged stress activates p38 MAPK and eventually induces cell death^20^. To examine the role of Drosha loss in the stress-induced cell death, we overexpressed wt and mt5 Drosha in SN4741 cells, applied 6-OHDA for 36 h, and measured cellular viability. This analysis showed that 6-OHDA led to a significant increase in the cleaved caspase 3. Overexpression of Drosha greatly reduced the level of activated caspase 3. Moreover, cells expressing mt5 Drosha had lower level of cleaved caspase 3 compared with the wt Drosha group (Fig. 4d). We further assessed the cellular viability by MTT and TUNEL assays and showed that enhancing Drosha level protected cells. Furthermore, mt5 Drosha offered cells better protection than wt Drosha (Fig. 4e, f). Thus, the degradation of Drosha may underlie 6-OHDA-induced loss of cell viability.

### Enhancing Drosha alleviated the DA neuronal loss in 6-OHDA-induced mouse model of PD

**Fig. 5:**
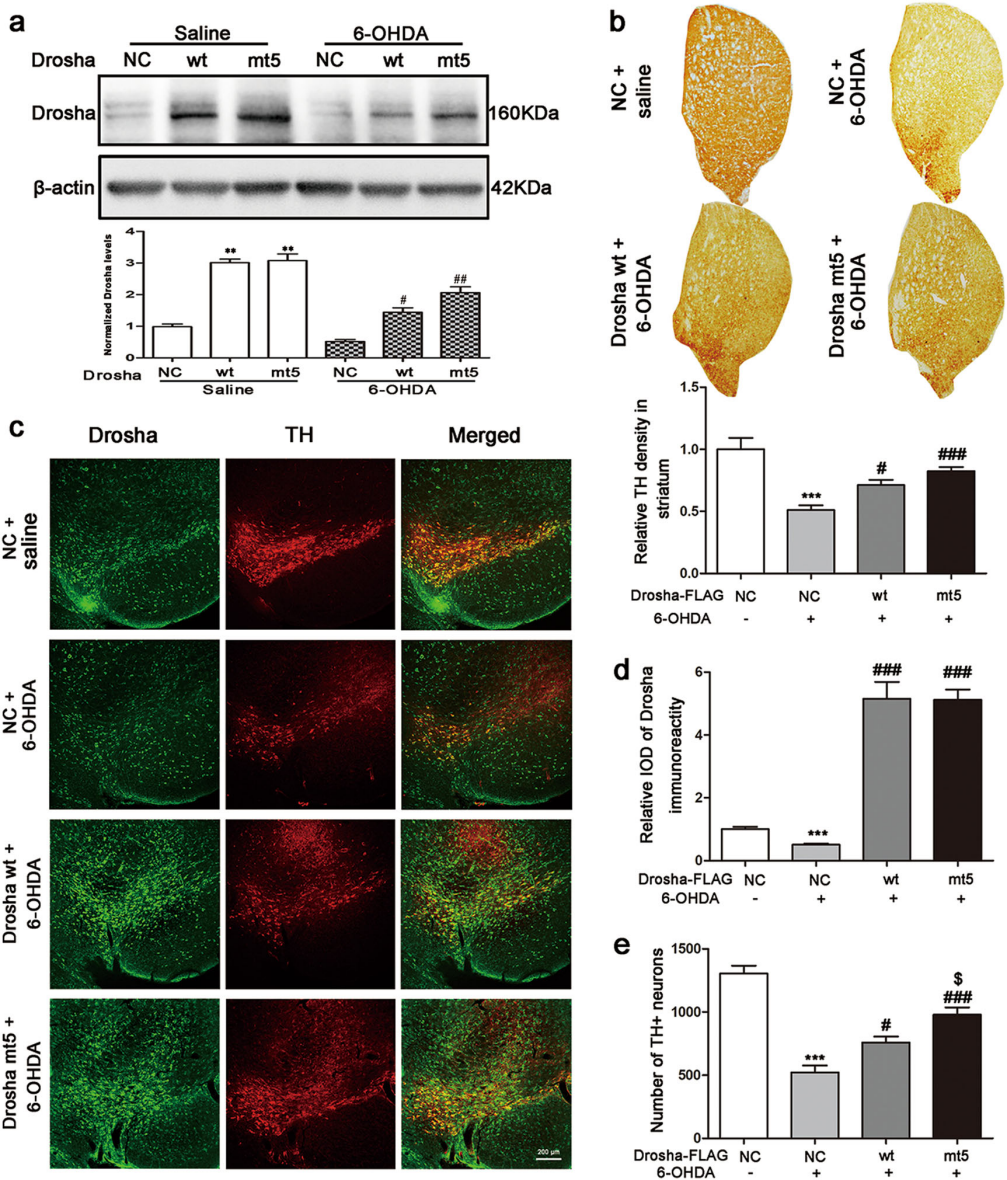
Enhancing Drosha alleviated the DA neuronal loss in 6-OHDA-induced mouse model of PD. **a** Drosha levels in SNc of PD mice after injection of negative control vector or adenovirus vectors expressing WT and mt5 Drosha. The adenovirus vectors were injected into the SNc of mice for 3 days before injection of 6-OHDA. Five days after 6-OHDA injection, the mouse brain was removed for immunoblot analysis. (ANOVA test followed by Tukey HSD, *P < 0.05, **P < 0.01, compared with the negetive control group. ^#^*P* < 0.05, ^##^*P* < 0.01 vs. negative control vector treated with 6-OHDA. ^$^*P* < 0.05 vs. WT vector treated with 6-OHDA, *n* = 3). **b** The TH-DAB immunostaining examined the dopaminergic terminal in striatum. Overexpression of Drosha correlated with the higher level of TH signal. (ANOVA test followed by Tukey HSD, ***P < 0.001, compared with the negetive control group. ^#^*P* < 0.05, ^###^*P* < 0.001 vs. negative control vector treated with 6-OHDA, *n* = 3). **c** Immunocytochemical analysis of the midbrain sections of PD mice received injection of adenovirus vectors expressing WT and mt5 Drosha. Restoring the level of Drosha protected the DA neurons from the toxicity of 6-OHDA. **d** The quantitative value of Drosha. (ANOVA test followed by Tukey HSD, ***P < 0.001, compared with the negetive control group. ^###^*P* < 0.001 vs. negative control vector treated with 6-OHDA, *n* = 3). **e** The number of TH-positive neurons. (ANOVA test followed by Tukey HSD, ***P < 0.001, compared with the vehicle control group. ^#^*P* < 0.05, ^###^*P* < 0.001 vs. control vector treated with 6-OHDA. ^$^*P* < 0.05 vs. WT vector treated with 6-OHDA, *n* = 3)

Since maintaining the level of Drosha attenuated the toxicity of 6-OHDA in vitro, we tested the effect in vivo in 6-OHDA-induced lesion of SNc DA neurons in mice. We injected the control or adenovirus expressing wt and mt5 Drosha in the SN region of 2 months old mice unilaterally and after 3 days, injected 6-OHDA for another 5 days. Western blot analysis of the SNc confirmed the increased level of Drosha following adenovirus-mediated expression in the saline group. 6-OHDA reduced the levels of both endogenous and overexpressed Drosha but mt5 Drosha was more resistant to 6-OHDA-induced degradation compared with wt Drosha (Fig. 5a). The TH-DAB immunostaining showed that 6-OHDA caused a significant DA terminal loss. Overexpression of Drosha correlated with a higher level of TH signal (Fig. 5b). Immunocytochemical analysis of the midbrain sections also confirmed that Drosha protected the TH-positive neurons from the toxicity of 6-OHDA. The number of remaining DA neurons also correlated with the Drosha level (Fig. 5c–e). These results demonstrate that enhancing the level of Drosha protects the SNc DA neurons from 6-OHDA-induced toxicity.

### Drosha ameliorated 6-OHDA-induced motor deficits in PD mice

**Fig. 6:**
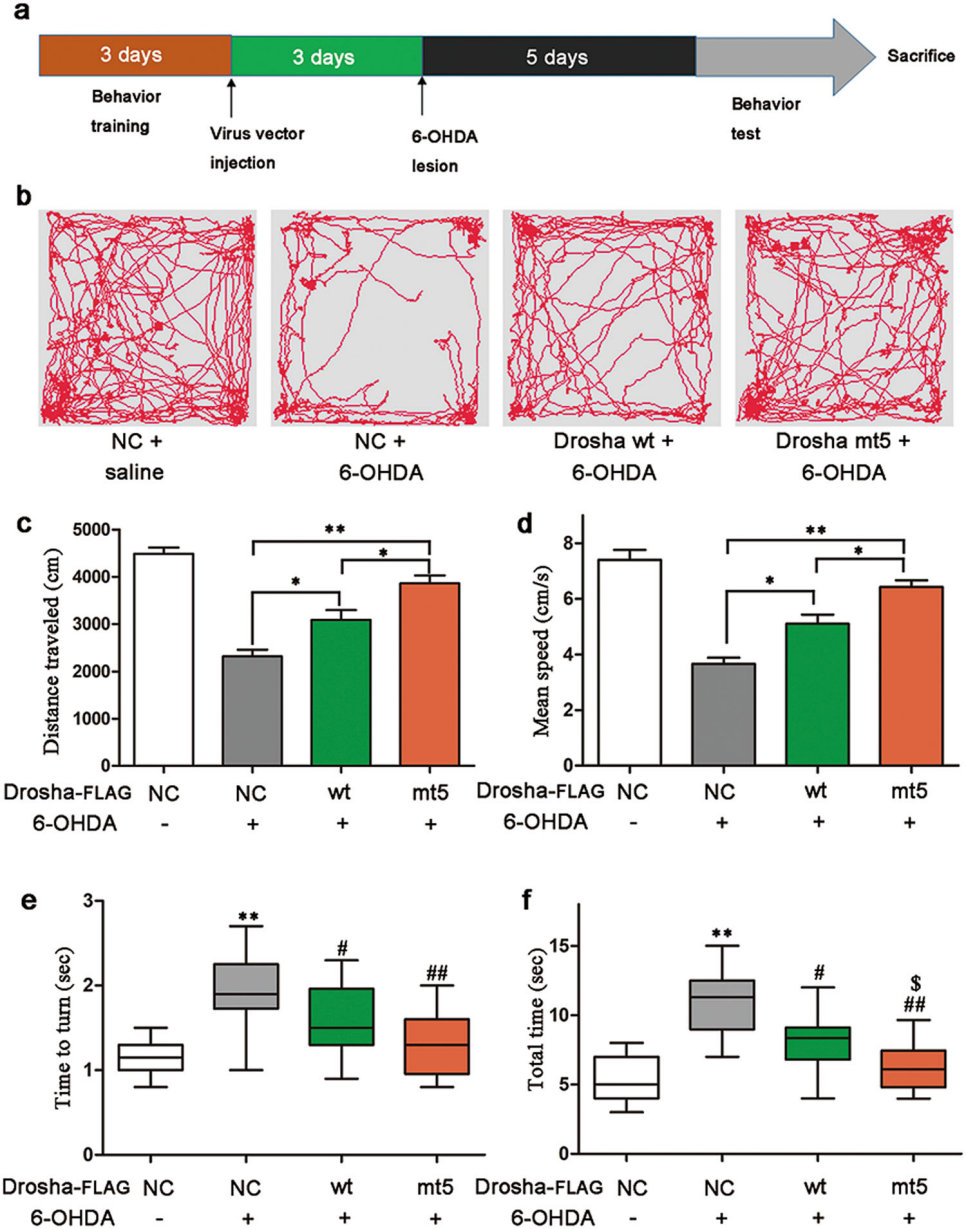
Drosha ameliorated 6-OHDA-induced motor deficits in PD mice **a** Experiments design of behavior tests. **b**–**d** Open-field parameters: trajectory chart, distance traveled (cm) and mean speed (cm/s). As described in methods, animals were trained before any treatment. The mice were recorded for 10 min in open-field chamber. (ANOVA test followed by Tukey HSD, *P < 0.05, **P < 0.01, *n* = 12). **e**, **f** The time to turn and total time of pole test. (ANOVA test followed by Tukey HSD, *P < 0.05, **P < 0.01, compared with the vehicle control group. ^#^*P* < 0.05, ^##^*P* < 0.01 vs. control vector treated with 6-OHDA. ^$^*P* < 0.05 vs. WT vector treated with 6-OHDA, *n* = 12)

Motor deficit is one of the principal clinical manifestations of PD. To test whether Drosha could relieve the motor deficits, we used the open-field and pole tests to assess the motor ability of PD mice. As showed in experiment design (Fig. 6a), the mice were placed in open-field chamber 10 min or trained on the pole for three consecutive days before receiving viral and 6-OHDA injection. Five days after 6-OHDA injection, mice were subjected to open field and pole tests. The results showed that the 6-OHDA caused a significant decrease in both distance and speed of movement in the open-field chamber. Overexpression of Drosha significantly attenuated these deficits (Fig. 6b–d). Similarly, pole test showed that overexpression of Drosha, shortened the time needed for mice to turn as well as the total time to descend compared with the control group (Fig. 6e, f). More importantly, mt5 Drosha was more effective than wt Drosha in ameliorating these motor deficits. These data indicate that augmenting Drosha effectively improves the motor deficits caused by 6-OHDA in mice.

## Discussion

One of the pathological hallmarks in PD is the gradual loss of DA neurons in the SNc. Although several factors attribute to the vulnerability of DA neurons including abnormal kinase activation, aggregation of misfolded protein, mitochondrial dysfunction and the miRNA dyshomeostasis, the exact etiology for the selective loss of DA neurons remains to be fully identified^21,22^. This lack of understanding contributes in part to the current PD treatment being aimed at symptom relief and not being able to reverse the course of the disease^23^. Our study uncovered a loss of Drosha in the 6-OHDA-induced cellular and animal models of PD. We found that 6-OHDA activated p38 MPAK and led to a p38 MAPK-dependent phosphorylation of Drosha, which caused Drosha degradation. Furthermore, restoring the level of Drosha alleviated 6-OHDA-induced cell death in vitro and the loss of the SNc DA neurons in vivo, highlighting Drosha as a critical factor in DA neuronal survival.

Phosphorylation appears to be a critical post-translational modification modulating the function of Drosha. For example, Drosha has been shown to be phosphorylated by GSK3β, which is reported to change Drosha subcellular localization and affect its activity^24,25^. In addition to phosphorylation, Drosha is also modified by ubiquitination. Drosha has been shown to be a substrate for ubiquitin E3 ligase. Ubiquitination of Drosha induced by mTOR and Mdm2 affects the level of Drosha and regulates miRNA biogenesis in response to energy deprivation^16^. In our current study, Drosha was phosphorylated by p38 MAPK under PD-related stress. Thus, post-translational modifications are the primary mode that tightly controls Drosha function.

As a class of powerful modulator of gene expression, miRNAs play a vital role in resisting stress^26–28^. Multiple stress signals associated with neurodegenerative conditions have been shown to alter miRNA profiles^29,30^. For example, p53 is involved in mediating stress response by enhancing the expression of certain primary miRNA transcripts via associating with DDX5, a co-factor for Drosha^31,32^. Activation of NF-κB has also been linked with the upregulation of certain miRNAs under stress conditions^33^. Our current study shows that PD associated stress may negatively modulate miRNA biogenesis at a global level by targeting Drosha, establishing Drosha inhibition as a direct mechanism of dysregulating miRNA biogenesis under pathogenic conditions associated with PD.

Modulating miRNA biogenesis has been implicated in both survival and death in various cell types and diseases^34–36^. Drosha is required for vascular smooth muscle cells survival by targeting multiple signaling pathways including phosphatidylinositol 3-kinase/AKT^37^. In testis development, mutation of Dicer is associated with a progressive loss of Sertoli cells, hinting that Dicer is necessary for Sertoli cell survival in mouse testes^38^. In our study, loss of Drosha is associated with 6-OHDA-induced toxic stress while enhancing Drosha is sufficient to protect the SNc DA neurons from 6-OHDA-induced death, suggesting that Drosha plays a role in the survival of the SNc DA neurons and its dysfunction may underlie the pathogenic process of PD.

Though Drosha is critical for DA neuron resistance to neurotoxin, the underlying mechanistic details remain unclear. Recently, specific conditional ablation of Dicer in DA neurons was reported to lead to the progressive loss of DA neurons with severe locomotor deficits^39^. Drosha with DGCR8 in the microprocessor cleaves pri-miRNAs into the pre-miRNAs and controls the initial step of miRNA biogenesis. It is likely that this regulatory function mediates the protective role of Drosha. This may involve specific individual miRNAs, such as miR-133b, miR-7, miR-184-5p, miR-153, and others, which are implicated in maintaining DA neuronal homeostasis and involved in the pathogenesis of PD^40–42^. For example, the negative-feedback loop between miR-133b and its transcription factor Pitx3 modulate the DA neuron differentiation^43^. α-synuclein protein is a key factor in the pathogenesis of PD and aggregation of α-synuclein is deleterious to DA neurons. MiR-7 has been reported to target α-synuclein mRNA and control the level of α-synuclein protein, leading to the increased resistance to oxidative stress^44^. Since degradation of Drosha signals a total dysfunction of pri-miRNA processing, loss of Drosha may lead to a dyshomeostasis of the global miRNA environment and sensitize DA neurons to stress or even trigger their death.

Recently, Drosha had been reported to regulate cellular activities through a miRNA independent mechanism. For example, Drosha may regulate human mesenchymal stem cells (hMSCs) cell cycle progression potentially by affecting ribosomal RNA (rRNA) processing or modulate neurogenesis by controlling Neurogenin 2 gene expression^45,46^. The non-canonical functions of nuclear Drosha and Dicer had been reported to include direct regulation of transcriptional initiation and termination as well as the processing of various types of RNA species^47^. Therefore, it is possible that Drosha augments cell survival via its non-canonical function. Further studies are needed to clarify this issue.

In summary, the present study reveals that neurotoxin 6-OHDA triggers a phosphorylation-dependent degradation of Drosha, which underlies 6-OHDA-induced toxicity. Thus, loss of Drosha function may be involved in the pathogenic process of PD. Our findings highlight the possibility of enhancing the function of Drosha as a potential therapeutic strategy to protect the SNc DA neurons in PD.

## Materials and methods

### Antibodies and regents

Antibodies and regents used in this experiment are all obtained commercially: Anti-Drosha antibody (ab12286), Anti-DGCR8 (ab191875) was purchased from Abcam (Cambridge, UK). Anti-TH antibody (P8984), anti-β-actin antibody (A5441), DAPI (D9542), 6-Hydroxydopamine hydrochloride (H4381), and SB203580 (S8307) were purchased from Sigma (St. Louis, MO, USA). Anti-phospho S/P (2325), Anti-phospho p38 (9211), anti p38 MAPK (8690), and anti-cleaved caspase3 (9664) were from Cell Signaling technology (Cambridge, MA, USA). Anti Flag (20543-1-AP) was purchased from Proteintech (Wuhan, China). Calpeptin (S7396) and MG132 (S2619) were purchased from selleck (Houston, USA). A TUNEL staining kit (QIA39) was purchased from Calbiochem (Boston, MA, USA). Vectastain ABC kit (PK-6101) was purchased from Vector Laboratories (Burlingame, CA, USA). DAB staning kit (CW0125) was purchased from CWBIO (Beijing, China). Thiazolyl blue (M8180) was purchased from Solarbio (Beijing, China).

### Animal and tissue preparations

Adult male C57BL/6 mice were purchased from the Experimental Animal Center of the Fourth Military Medical University. They were housed in standard cages with 12 h light/dark cycle and free access to food. We made efforts to minimize animal suffering and reduce the number of animals used according to the Guidelines for Animal Care and Use of the Fourth Military Medical University (Xi’an, People’s Republic of China). Animals were killed 5 days after 6-OHDA injection. They are anesthetized (10% chloralhydrate, 35 mg/kg, i.p.) and transcardially perfused with 0.9% NaCl solution for 3 min. The brains were immediately removed to prepare for Western blot analysis. For immunofluorescent assay, they were continued to be perfused with cold 4% paraformaldehyde in phosphate buffer for 1 h. Then the brains were removed and placed in 15% sucrose at 4 °C for 2 days. Serial cryostat sections were collected with a microtome (CM1950, Leica, Wetzlar, Germany)^48^.

### Plasmid construction and adenovirus generation

The plasmids have been described previously^17^. To create the AV-Drosha vector, the DNA sequences corresponding to wt and mt5 Drosha were subcloned from the pcDNA3.1-Drosha vector into the multiple cloning site of the AV vector. We used DNA sequencing analysis to validate the integrity of the AV-Drosha construct. Viral vectors were packaged by Obio Technology (Shanghai, China).

### Cell culture

SN4741, a mouse midbrain DA neuronal cell line, were cultured in the Dulbecco modified Eagle’s medium supplemented with 10% fetal bovine serum (FBS), 1% d-glucose, and 120 mM l-glutamine with 5% CO_2_ at 33 °C. A total of 60–70% confluence was suitable for experiments. SN4741 cells were transfected with wt and mt5 Drosha using Lipofectamine 2000 (Thermo Fisher Scientific) according to the manufacturer’s protocol.

### Stereotaxic surgery

C57BL mice (8–10 weeks of age) were anaesthetized using chloral hydrate (35 mg/kg, i.p.). The stereotaxic instrument (RWD 68901, Shenzhen, China) was used with a 1 μl Hamilton syringe to inject the 6-OHDA or adenovirus directly into the SNc in relation to Bregma (mm): 1.1 ML, 3.10 AP, 5.6 DV^18,49^. The lambda and bregma sutures were aligned in the same horizontal plane. Drillinged a hole in the skull, 1 μl of AV viral particles (9 × 10^10^ pfu/ml) or 0.3 μl 6-OHDA (20 μM) were injected into the brain. The regent was injected at a rate ∼200 nl/min. The needle was left in place for 5 min before being slowly withdrawn from the brain. The skin over the injection site was closed by suturing. The animal was allowed to recover for 3 days to maximize viral gene expression before behavioral testing, euthanization, or any treatment paradigm.

### Immunofluorescence

Brain sections and cell slides were fixed with 4% formaldehyde solution followed with 0.5% Triton X-100 for 40 min. Then blocked samples with 5% bovine serum albumin (BSA) (Sigma-Aldrich) in PBS for 30 min. Samples were incubated with primary Anti-Drosha antibody (ab12286) combined with anti-TH antibody (P8984) overnight at 4 ℃. Samples are washed three times with PBS and incubated with secondary antibodies at 27 ℃ for 2 h. Samples were counterstained with DAPI for 10 min and photographed using a confocal microscope (Nikon A1).

### Co-immunoprecipitation and immunoblotting

The mouse brains or cells were lysed in RIPA buffer (P0013C, Beyotime, Shanghai, China) containing protease cocktail inhibitor (539134, Millipore Corporation, Bedford, MA, USA) and Phosphatase inhibitor (524625, Millipore Corporation, Bedford, MA, USA). The protein levels were quantified using Pierce BCA Protein Assay Kit (23227, Thermo scientific). Co-immunoprecipitation was performed with Drosha or Flag antibody and protein A/G Sepharose (Santa Cruz Biotechnology, Boston, MA, USA). The cell lysate should incubate with the antibody more than 16 h at 4 ℃. After that, incubate with protein A/G Sepharose for 6 h. The immunocomplexes were then washed with RIPA buffer for three times, and the samples were prepared by adding a sample-loading buffer. We separated proteins by sodium dodecyl sulfate polyacrylamide gel electrophoresis and transferred to polyvinylidene fluoride membranes (Millipore Corporation, Bedford, MA, USA). Then block the membranes with 5% fat-extracted milk at room temperature for 2 h. Next, incubated membranes with primary antibodies overnight at 4 °C. Then washed three times with Tris-buffered saline Tween and incubated with secondary antibody. Protein bands were visualized using ECL.

### DAB immunostaining

DAB immunostaining was performed on striatum^50^. We immersed sections in 3% H2O2 for 30 min at room temperature. Next, 30-mm sections were incubated with anti-TH antibody overnight at 4 ℃. Then used the Vectastain ABC kit followed incubation with biotinylated secondary antibody. The peroxidase reaction product was detected using 3ʹ3ʹ-diaminobenzidine-tetrahydrochloride (DAB; CWBIO). Washed with PBS three times, sections were dehydrated in a graded series of ethanol and immersed in xylene.

### **C**ell counting and intensity analysis

Using the medial terminal nucleus (MTN) of the accessory optic tract as its medial border, we quantified the number of TH-positive neurons in the entire pars compacta^51^. We collected the cryosections and every fourth cryosection was used for counting the DA neurons. The intensity of Drosha and tyrosine hydroxylase were analyzed by using Image J in the substantia nigra and striatum. We sampled 28 × 28 pixel area in 30 images from 5–8 consecutive sections. The values are average intensity above background ± s.e.m^52^.

### MTT assay

Almost 5 × 10^3^ cells were seeded in every well of 96-well plates and incubated at 33 °C with 5% CO_2_. After treatment with 6-OHDA, 20 μl of 3-(4,5-dimethylthiazol-2-yl)-2,5-diphenyltetrazolium bromide (MTT) (5 mg/ml) were added to each well, and then incubated the plate for 4 h. Removed supernatant, added 150 μl of dimethylsulfoxide (DMSO) (Millipore, Bedford, MA, USA) to each well and mixed thoroughly for 16 min. Measured the optical density (OD) at 490 nm in SpectraMax M2 (Molecular Devices, California, USA).

### TUNEL staining

According to manufacturer’s instructions, FragEL™ DNA Fragmentation Detection Kit was used for terminal deoxynucleotidyl transferase-mediated dUTP nick end labeling (TUNEL) staining. We used the Image J software to count the positive cells^53^.

### Behavior test

We collected and analyzed activity data by EthoVision XT 8.5. The dimensions of the activity chamber (San Diego Instruments) were 50 cm by 50 cm by 38 cm. Mice were placed daily inside the chamber for 10 min before treatment for 3 days. Ten-min test sessions were recorded for each mouse to record open-field activities^54^. After each record, we clean the chamber with 70% ethanol to clear the smell left. The pole test was widely used to examine bradykinesia and motor coordination in mice model of PD. We put the mice facing upward at the top of a wooden pole (50 cm long and 1.5 cm in diameter). The mice were trained to turn to orient downward and traverse the pole into their homecage before any treatment. At indicated time after surgery, we tested the amount of time to turn to orient downward and the total time used on the pole (from the time that mouse is placed on the pole until it reaches the base of the pole in the homecage)^55^. Each mouse was tested for five time and median data was used for further analysis.

### Statistical analyses

Data were expressed as mean ± standard error of the mean (SEM) from at least three independent experiments. Data were analyzed by Student’s *t*-test or one-way analysis of variance (ANOVA) followed by Tukey post hoc analysis as appropriate. Statistical analyses were carried out using SPSS 19.0 (SPSS, Michigan Avenue, Chicago, IL, USA). A value of *P* < 0.05 was considered statistically significant.

## Electronic supplementary material

SUPPLEMENTAL data

supplementary data figure legend
