# Supplementary figures and images for: Loss of Drosha underlies dopaminergic neuron toxicity in models of Parkinson’s disease
Source: Cell Death Dis. 2018 Jun 7;9(6):693. doi: 10.1038/s41419-018-0716-5 (PMC5992196; doi:10.1038/s41419-018-0716-5)

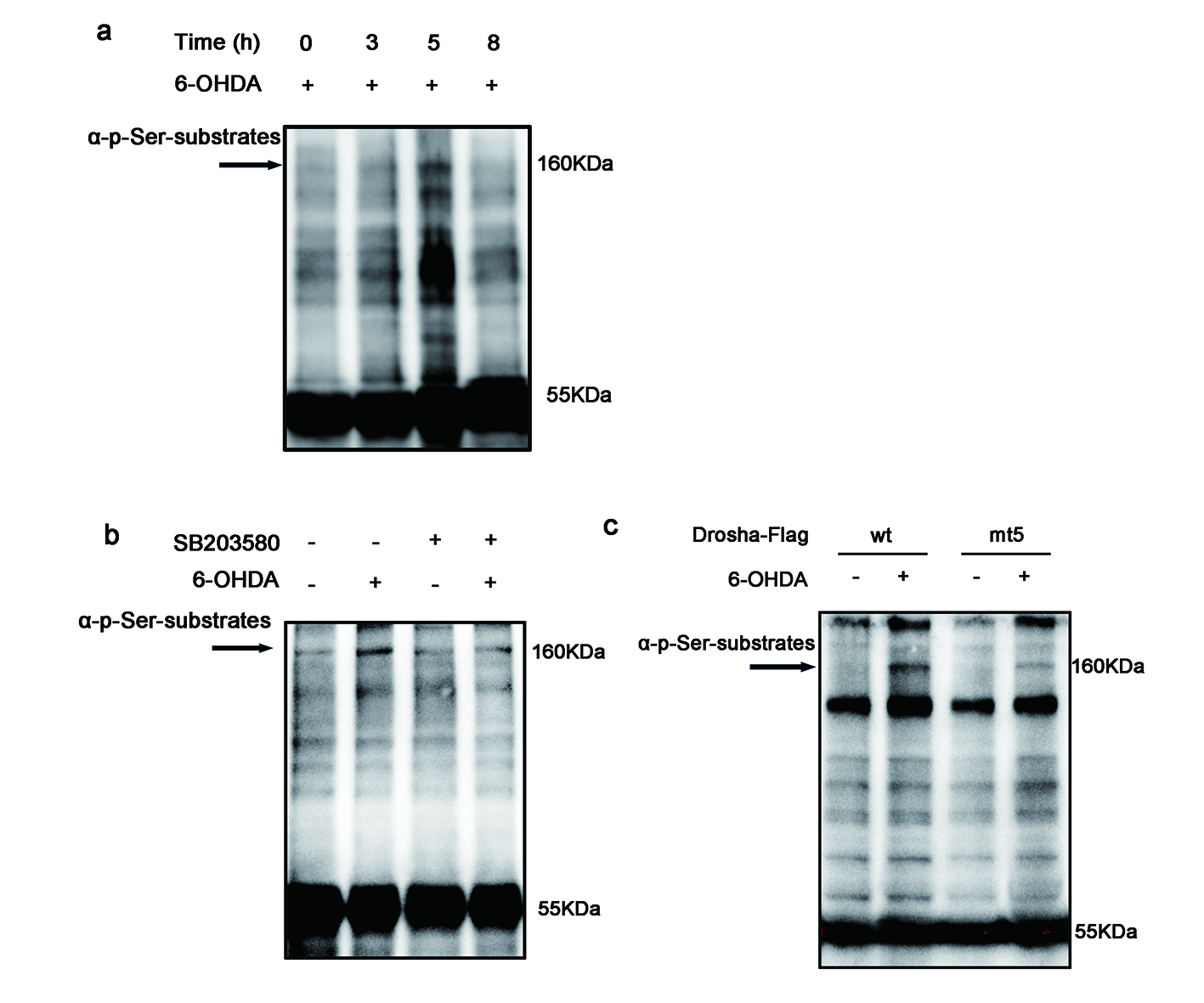

Supplement: Supplementary file 1 — SUPPLEMENTAL data [file 41419_2018_716_MOESM1_ESM.tif]
