# Supplementary material for: Loss of Drosha underlies dopaminergic neuron toxicity in models of Parkinson’s disease
Source: Cell Death Dis. 2018 Jun 7;9(6):693. doi: 10.1038/s41419-018-0716-5 (PMC5992196; doi:10.1038/s41419-018-0716-5)
Supplement: Supplementary file 2 — supplementary data figure legend [file 41419_2018_716_MOESM2_ESM.docx]

**Supplementary figure legend**

**(a)** The full blot of Figure 2(b)

**(b)** The full blot of Figure 2(c)

**(c)** The full blot of Figure 4(b)
